# Genome Damage in Rats after Transplacental Exposure to *Jatropha dioica* Root Extract

**DOI:** 10.1155/2019/2962950

**Published:** 2019-11-03

**Authors:** Gabriela Morales-Velazquez, Blanca Patricia Lazalde-Ramos, Belinda Claudia Gómez-Meda, Guillermo Moisés Zúñiga-González, Yveth Marlene Ortiz-García, Rosalinda Gutiérrez-Hernández, Celia Guerrero-Velazquez, Susana Vanessa Sánchez de la Rosa, Ana Lourdes Zamora-Perez

**Affiliations:** ^1^Instituto de Investigación en Odontología, Centro Universitario de Ciencias de la Salud, Universidad de Guadalajara, Guadalajara, Jalisco, Mexico; ^2^Doctorado en Farmacología, Centro Universitario de Ciencias de la Salud, Universidad de Guadalajara, Guadalajara, Jalisco, Mexico; ^3^Maestría en Ciencias y Tecnología Química, Unidad Académica de Ciencias Químicas, Universidad Autónoma de Zacatecas, Zacatecas, Mexico; ^4^Instituto de Genética Humana “Dr. Enrique Corona Rivera” Departamento de Biología Molecular y Genómica, Centro Universitario de Ciencias de la Salud, Universidad de Guadalajara, Guadalajara, Jalisco, Mexico; ^5^Laboratorio de Mutagenesis, Centro de Investigación Biomédica de Occidente, Instituto Mexicano del Seguro Social, Guadalajara, Jalisco, Mexico; ^6^Licenciatura en Nutrición, Unidad Académica de Enfermería, Universidad Autónoma de Zacatecas, Zacatecas, Mexico

## Abstract

*Jatropha dioica* is traditionally used owing to its antiviral, antifungal, and antimicrobial properties. But, toxicological information regarding *J. dioica* root total extract is currently limited. The aim of this work was to evaluate in a rat model, the transplacental genotoxicity effect of *J. dioica* aqueous root total extract. Three different *J. dioica* aqueous root total extract doses (60, 100, and 300 mg/kg) were administered orally to Wistar rats during 5 days through the pregnancy term (16–21 days). Pregnant rats were sampled every 24 h during the last 6 days of gestation, and pubs were sampled at birth. *Genome* damage in dams and their newborn pups transplacentally exposed to *J. dioica* was evaluated by *in vivo* micronuclei assay. We evaluated the frequency of micronucleated erythrocytes (MNE), micronucleated polychromatic erythrocytes (MNPCE), and polychromatic erythrocytes (PCE) in peripheral blood samples from pups and MNPCE and PCE in pregnant rats. No genotoxic effect was observed after oral administration of the three different doses of aqueous root total extract of *J. dioica* in pregnant or in their newborn pubs, after transplacental exposure. A significant decrease in PCE frequency was noted in samples from pubs of rats treated with the highest dose of *J. dioica* extract. The aqueous total root extract of *J. dioica* at the highest dose tested in our research do have cytotoxic effect in pups transplacentally exposed to this plant extract. Moreover, neither a genotoxic nor a cytotoxic effect was observed in pregnant rats. In the present work, there was no evidence of genome damage in the rat model after transplacental exposure to *J. dioica* aqueous root total extract.

## 1. Introduction

The use of traditional medicine against disease, which includes the use of medical plants extracts and teas, is widely spread in population and serves as a guide for scientists to support new drug development and validate the medical properties and safety of plants used in folk medicine [1, 2].

*Jatropha dioica* is a species of flowering plant in the spurge family, Euphorbiaceae, native to Mexico as far south as Oaxaca and United States, in Texas [3, 4]. *J. dioica* commonly known as *“sangre de drago”* (“dragon's blood”) has a colorless juice, which becomes darker when in contact with air [5]. Extracts from different parts of the *Jatropha* plant, such as the leaf, stem, bark, and roots, have been used in Mexican herbal medicine since the prehispanic era. This plant presents many positive health effects including antiviral, antifungal, and antimicrobial activities, and the root of the plant is chewed to treat buccal diseases, for example, in tooth mobility [5]. Some species of *Jatropha* have been demonstrated, in methanolic extracts of the rhizomes, to activate anti-inflammatory activity in rats with a dose of 200 mg/kg body weight [6]. Furthermore, the hydroalcoholic extracts of dragon's blood rhizomes present antioxidant and antimicrobial activities at a concentration of 50 mg/mL against fungi and against Gram-positive and Gram-negative bacteria [7, 8]. The antioxidant and antimicrobial properties of *Jatropha dioica* are related mainly to its content of phenolic compounds [9]. *J. dioica* is a rich source of phytochemicals such as alkaloids, lignans, cyclic peptides, and terpenes that are related with a broad range of biological activities [10] such as antiviral [7], antifungal [7, 11], and antimicrobial [7]; furthermore, the hyperglycemic and chemopreventive effect of *J. dioica* has also been described [12].

Regardless of the frequent use of natural products, there are lack of studies that provide information about the safe use of medicinal plants, since plants could present a therapeutic potential, but also adverse effects [13].

A few studies have evaluated the potential toxicity effect of *J. dioica*. Oliveira Simone et al. [14] performed an *in vitro* evaluation by means of the colorimetric MTT reduction assay (3-(4,5-dimethylthiazol-2-yl)-2,5-diphenyl tetrazolium bromide) that measures only living cells, and the authors reported low cytotoxic effect in an ethanolic and aqueous extract from leaves and roots of *J. dioica* in mouse fibroblast cells. Silva-Belmares et al. [7] observed no cytotoxicity in a hexane extract of *J. dioica* root in human hepatocyte cells, opossum kidney epithelial cells, and pig epithelial cells.

However, in just one report found in the literature, it evaluated the genotoxic and cytotoxic effect in vivo of *J. dioica* aqueous extract. Araujo-Espino et al. [15] detected neither a genotoxic nor a cytotoxic effect of *J. dioica* aqueous extract in mouse peripheral blood by the micronucleus (MN) assay.

Numerous tests are used to evaluate genome damage or genotoxic effect of chemical compounds and their natural products, including the MN assay. The in vivo evaluation of DNA damage by means of the MN assay is the primary test within a series of assay that evaluate genotoxicity and is suggested by the regulatory agencies worldwide as a part of product safety evaluation [16].

The micronuclei are chromosomal fragments or whole chromosomes that spontaneously or as a result of clastogenic or aneuploidogenic agents are excluded from the nucleus in mitosis [17]; therefore, MN indicates loss of DNA in the cell nucleus.

Genome damage in newborns after transplacental exposure to xenobiotics is unusual and poor for risk assessment. Thus, based on all the pharmacological properties of *J. dioica* in this work, we evaluate whether or not *J. dioica* aqueous root total extract induces the effect on DNA fragmentation or chromosomal loss during the gestational period. However, transplacental genotoxicity has not been evaluated in the offspring exposed to *J. dioica* aqueous root total extract, which would show whether compounds administered to the mother during pregnancy could be placental transferred via the placenta during the development of rat pups and thus produce a genotoxic effect to the offspring [18].

## 2. Materials and Methods

### 2.1. Animals

Three-month-old female Wistar rats (average weight 205.10 g ± 10.75 g) were studied. All animals were healthy and were provided by the animal facilities of the *Centro de Investigación Biomedica de Occidente, Instituto Mexicano del Seguro Social* (CIBO-IMSS) in, Guadalajara, Jalisco, Mexico. Rats were housed in polycarbonate cages in windowless rooms, with automatic temperature control (22 ± 2°C) and lighting (lights on at 07:00 and off at 19:00 h) and maintenance of relative humidity (50 ± 10%). Animals received standard laboratory pellet food (Purina, Mexico) and tap water *ad libitum*.

### 2.2. Plant Material

The *J. dioica* root used in this study was collected in June 2014 and provided by *Laboratorios DEMIR S.A. de C.V*., in Zacatecas, Mexico. The dry roots were labeled with the number 30036 by the *Centro Interdisciplinario de Investigacion para el Desarrollo Integral Regional of the Instituto Politecnico Nacional (*CIIDIR-IPN) herbarium unit in Durango, Mexico.

### 2.3. Preparation of the Aqueous Total Extract of the Plant

The dry roots of the plant were ground into a fine powder. The powder was dissolved at a proportion of 1 g per 10 mL of water. The mixture was placed under mechanical stirring during 2 h at a temperature of 65 ± 5°C, and then it was filtered. The filtrate was taken to dryness by means of freeze-drying or lyophilization. The samples were stored at a refrigerated temperature of 4°C for further use. The aqueous root total extract of *J. dioica* contains alkaloids, flavonoids, saponins, carbohydrates, and reducing sugars based on a phytochemical analysis [12].

### 2.4. Mating

**Figure 1:**
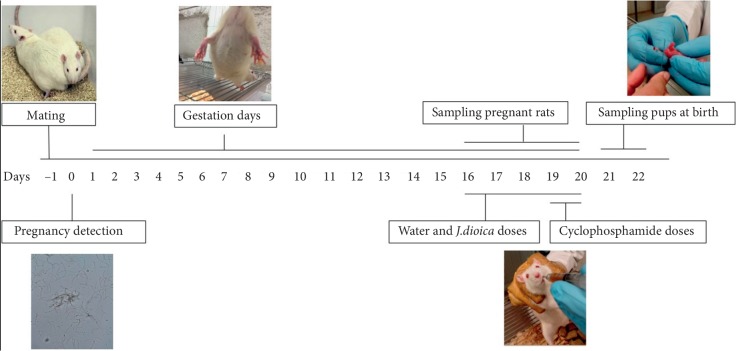
Schematic representation of transplacental MN assay.

Twenty-five female rats were mated with males in cages containing three females and one male. Each female rat was flushed daily with a vaginal wash of 0.1 ml of sterile water using an adjustable-volume pipettor, and the contents were smeared onto clean slides, which were analyzed without stain using a light microscope. The day of the initial identification of sperm was established as the first day of the pregnancy after which female rats were housed in individual cages, and a dose administration schedule was assigned for each rat [18, 19] (Figure 1).

### 2.5. Study Groups

The *J. dioica* aqueous root total extract doses selected were based on previous reports [12, 15]. Rats were randomly distributed into five groups (five rats per group; each rat was housed in an individual cage): Group 1: negative control, rats received sterile water; Group 2: *J. dioica* low dose, 60 mg/kg; Group 3: *J. dioica* middle dose, 100 mg/kg; Group 4: *J. dioica* high dose, 300 mg/kg; and Group 5: positive control, 60 mg/kg of cyclophosphamide (CP). The doses were administered orally with an esophageal cannula to each rat once daily for five days throughout the final stage of the gestation period (days 16 to 20 of the gestation process) [18, 19]. The dose of CP was administered in two doses of 30 mg/kg on days 19 to 20 of the gestation process. All doses were administered at a volume of 0.1 ml/10 g of weight.

### 2.6. Sample Preparation and Micronucleus Analysis

Blood samples were taken from pregnant adult rats by means of a small excoriation at the tip of the tail to perform smears in duplicate for each rat. Samples were collected before the administration of the first dose (basal sample) and every 24 h for 6 days at 0, 24, 48, 72, 96, and 120 h before each administration [20]. At birth (from day 21 and 22), six pups per rat were selected randomly and weighed, and a drop of blood was obtained from each pup from the tip of the tail to make two smears on precoded slides.

**Figure 2:**
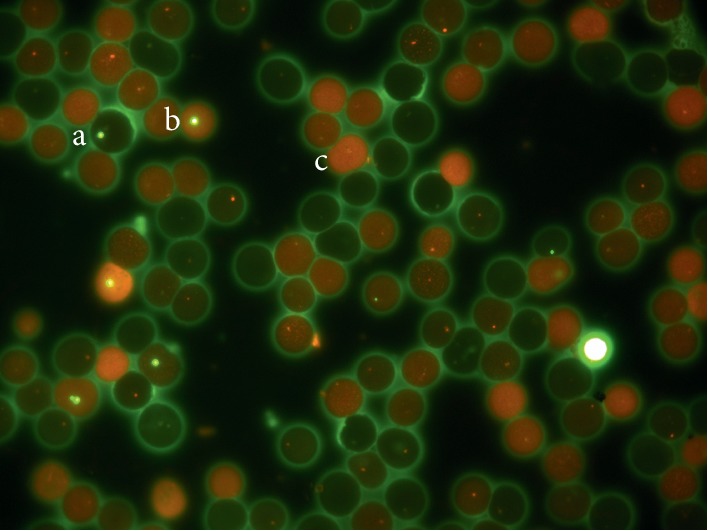
MNE (a), MNPCE (b), and PCE (c) from rat peripheral blood. Oil immersion objective 1000×, acridine orange stain.

All samples were air-dried, fixed in absolute ethanol for 10 minutes, and stained with acridine orange [21]. For manual analysis, an Olympus microscopy CX31 equipped with epifluorescence and an oil-immersion objective (100X) was used. For the pups, the number of micronucleated erythrocytes (MNEs) was counted in 10,000 total erythrocytes (TEs: polychromatic and normochromatic erythrocytes); the number of micronucleated polychromatic erythrocytes (MNPC) was counted in 1,000 polychromatic erythrocytes (PCEs), and the number of PCEs in 1,000 TEs was also determined as a control system since a reduction in PCE number reveals bone marrow toxicity (Figure 2). For adult rats, the number of MNPCEs was counted in 3,000 PCEs, and the proportion of PCEs was counted in 1, 000 TEs [18, 19]. Adult rats have very few MNEs in peripheral blood; therefore, the MN assay must be performed using bone marrow and/or by counting MNPCEs in peripheral blood [22].

### 2.7. Statistical Analysis

Data (‰) are expressed as the mean ± standard deviation of MNE, MNPCE, and PCE frequencies. Normality distribution was determined using the Kolmogorov–Smirnov test. For newborn rats, the dams were used as the experimental unit (*n* = 6/group). One-way ANOVA, followed by Dunnett's test for multiple posthoc pairwise comparisons versus the appropriate control, were employed to correct the significance values for intergroup analysis. Intragroup comparisons were made between each treatment group and their respective basal value (0 h) by means of repeated measures ANOVA, followed by a Bonferroni posthoc test for multiple comparisons. A *P* value less than 0.05 was considered significant, and all results were evaluated using the Statistical Program for Social Sciences (SPSS v. 20, IBM Co., Armonk, NY, USA).

### 2.8. Ethical Considerations

This study was approved (registration no. CII9/2016) by the Research Ethics Committee of the University of Guadalajara. All experiments were performed in accordance with the guidelines for the use and care of research animals specified in the regulations and national norms (official Mexican standard NOM-062-ZOO-1999) and of the International Institutes of Health for the humane treatment of research animals [23, 24]. At the end of the experimental period, animals were deeply anesthetized with an intraperiotoneal injection of ketamine (100 mg/kg) which was followed by sacrificing the rats with intracardiac injection of potassium chloride (0.05 ml) as per the guidelines of the Institutional Animal Ethics Committee (IAEC). Management of animal use followed the principles and guidelines approved by the Guide for the Care and Use of Laboratory Animals, while euthanasia followed the CONCEA Euthanasia Practice Guidelines.

## 3. Results

In the present study, the mean weight of pregnant rats before treatment (at day 16 of pregnancy) was 241.26 ± 22.53 g. The average number of offspring per litter was 6.61 ± 3.04 pups, and the average weight of the pups at birth was 5.68 ± 0.66 g.

**Figure 3:**
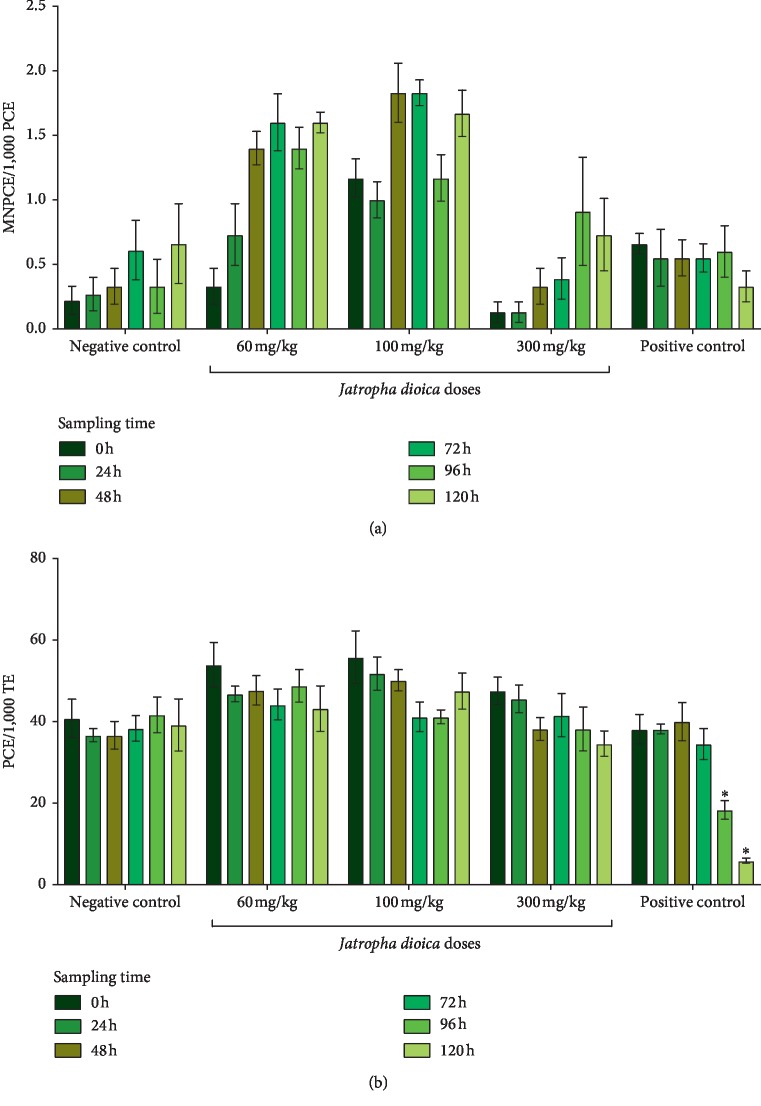
Proportion of MNPCE and PCE in pregnant rat peripheral blood in the study groups. Mean values are expressed as columns, and error bars represent standard deviation. Intragroup comparisons were performed between baseline samples (0 h against the following sampling times: 24, 48, 72, 96, and 120 h. No statistically significant increases were observed in MNPCE and PCE numbers at any sampling time. PCE: polychromatic erythrocytes; MNPCE: micronucleus polychromatic erythrocytes; total erythrocytes.

In pregnant rats, the statistical significance of the intra- and intergroup comparisons of MNPCE and PCE from the different study groups are shown in Table 1. Regarding the intra- and intergroup comparisons of MNPCE frequencies in pregnant rats, no significant changes were observed in any of the study groups (Figure 3). When PCE values were analyzed in the intragroup comparisons, a significant decrease at 96 h (*P*=0.007) and 120 h (*P*=0.005) was observed in the positive control group. Moreover, in the case of intergroup comparisons, a significant increase in PCE frequency was observed in the group treated with the middle dose of *J. dioica* aqueous root total extract (100 mg/kg) at 24 h (*P*=0.002) and 48 h (*P*=0.04), whereas the positive control group exhibited significant differences at 96 h (*P*=0.001) and 120 h (*P*=0.001), due to a decrease in the number of PCE (Figure 3).

**Figure 4:**
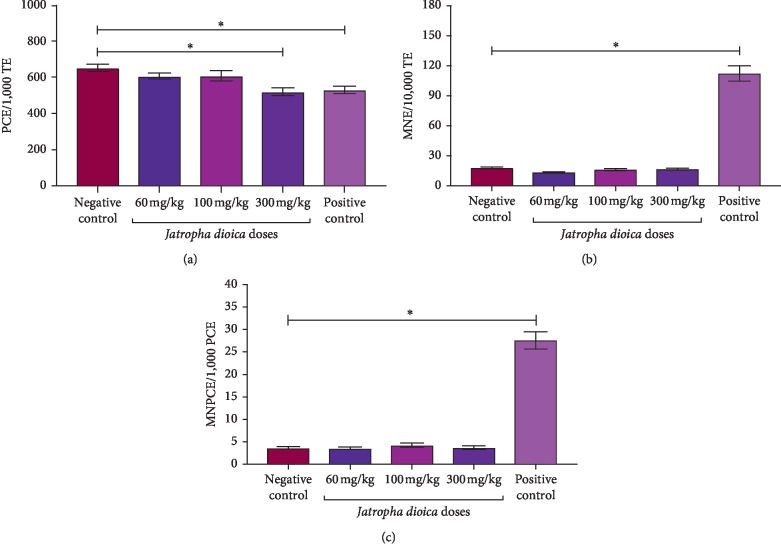
Proportion of PCE, MNE, and MNPCE from pups of the pregnant rat in the study group. Mean values are expressed as columns, and error bars represent standard deviation. Intergroup analysis by means of one-way ANOVA and Dunnet *t*-test posthoc for multiple comparisons. PCE: polychromatic erythrocytes; MNE: micronucleus erythrocytes; MNPCE: micronucleus polychromatic erythrocytes; total erythrocytes. ^*∗*^*P* < 0.001.

In pups of rats, the statistical significance of the intergroup comparisons of PCE, MNE, and MNPCE from the different study groups are shown in Table 2. In the pups exposed to *J. dioica* aqueous root total extract during the gestational period, a significant increase was observed in MNE (*P*=0.001) and MNPCE (*P*=0.001) positive control values when compared with the negative control, and no differences were observed when the negative control group was compared with the study groups treated with *J. dioica* aqueous root total extract (Figure 4). However, PCE values decrease significantly in the positive control group and in the group exposed to the highest dose (300 mg/kg) of *J. dioica* aqueous root total extract (*P*=0.001) (Figure 4). In addition, the weight of pups and number of offsprings at birth were analyzed, and a significant decrease in the weights of neonates (*P*=0.001) in the positive control group compared with the negative control group was observed (Table 3). No significant differences were found in the number of offspring at birth in any of the study groups.

## 4. Discussion

Genotoxicity assays are used to determine those compounds that can interact with the genetic material. When genotoxic compounds interact with DNA, this may lead to chromosomal damage that affects the DNA structure and thus produce permanent changes in the cell [25].

To date, there is no information about the possible genotoxic effect of *J. dioica*, during the gestational period. In this work, we evaluated the genotoxic effect of three different doses of *J. dioica* aqueous root total extract, administered orally to pregnant rats, and we assessed the effect on pups, since the neonate rat is a very sensitive model for detecting genotoxicity by the transplacental MN assay [18]. This model can evaluate whether the agent administrated to the mother could cause harmful effects on the fetus because of an increase in the MNE observed in the peripheral blood of neonates. This MNE increase provides information regarding the possible genotoxicity and teratogenic potential of the test agent [18].

The plant *J. dioica* is a folk medicine, and the roots of the plant (20 g approximately) are cooked in hot water, and the liquid is applied locally to treat hair loss, dandruff, strokes, small wounds, acne, and also for other skin conditions. Also, *J. dioica* is traditionally used in the treatment of dental issues such as gingivitis, loose teeth, bleeding gums, and toothache, for example, root mastication to prevent tooth mobility [5].

As expected, the MNE and MNPCE frequencies increased in the positive control group compared with the negative control group since CP is considered a micronucleogenic agent, and it is used as a positive control in genotoxicity tests [21]. Furthermore, PCE frequency decreased significantly due to the cytotoxic effect of CP [18, 26]. Nevertheless, the *J. dioica* aqueous root total extract treated groups did not show MNE nor MNPCE increase frequencies in pregnant rats or in the offsprings. Our results are similar to those reported by Araujo-Espinoza et al. [15], where evaluated *in vivo* the genotoxic and cytotoxic effect of four different doses of *J. dioica* aqueous root total extract administered orally to mice using the erythrocyte MN assay did not show either a PCE decrease or an increase in PCMNE and MNE.

On the other hand, the proportion of PCE/TE is considered as a marker for the system to establish a compound cytotoxicity because of its myelosuppressive effect [25]. In the present work, a significantly diminished proportion of PCE compared with the negative control group was observed in the pups of pregnant rats exposed to the highest dose of *J. dioica*. This result can be explained by the presence of different phytochemical compounds of the plant [14]. For example, riolozatrione tricyclic diterpene and diterpene epoxide citlalitrione have been isolated from the roots of *J. dioica*, and it has been described that most of diterpenes could exhibit cytotoxic activities in vitro [27]. This cytotoxic effect observed as a decrease in PCE frequency in neonates of pregnant rats exposed to the highest dose of *J. dioica* is in accordance with that reported by Oliveira Simone et al. [14] since they evaluated in vitro the cytotoxic activity of the aqueous and ethanolic extract from leaves and roots of *J. dioica* in mouse fibroblast cells by the colorimetric test of bromide reduction, and the authors report a low cytotoxicity of the aqueous and ethanolic extract of *J. dioica*. Zhang et al. [28] reported that many species of the Euphorbiaceae family have toxic components, which are capable of destroying normal cells in mammals. Nevertheless, Silva-Belmares et al. [7] indicated that Mexican *J. dioica* species do not have that effect.

Contrary to our results, Silva-Belmares et al. [7] reported a nonsignificant cytotoxicity activity of the hexane extract of *J. dioica* extract in Chang cells (human hepatocyte cells), OK (opossum kidney epithelial cells), and LLCPK-1 (pig epithelial cells) by the colorimetric MMT test of bromide reduction.

Considering the weight and number of offsprings, in the present study, the newborns exposed to CP during the gestational period show a statistically significant decrease in weight compared with the negative control group due to the cytotoxic effect of CP [21, 26, 29].

The genotoxic evaluation of medicinal plants by means of an *in vivo* transplacental model, including *J. dioica*, might be considered since medicinal plants not only present curative effects but may also show toxic effects associated with their phytochemical compounds [30]. This plant presents antitumoral, antimicrobial, and antioxidant properties [31]; however, it has also been reported that the plant seeds produce toxic effects if they are consumed as infusions. The main toxic effects are related to the damage at DNA level in cells; however, it has been demonstrated that such effects can be eliminated if the concentrations used are appropriate to take care of the toxicological aspects in order to preserve the biological effects [12].

Lastly, the fetus can be exposed to many different chemicals during pregnancy, including genotoxic compounds, drugs, or food carcinogens through placental transfer [32]. Therefore, it is important to know whether compounds to which mothers are exposed would damage their own genetic material and/or their offsprings.

## 5. Conclusion

In conclusion, no evidence of genome damage was observed when the doses of *J. dioica* aqueous root total extract administered orally to pregnant rats during the final phase of gestation. However, the highest dose of *J. dioica* aqueous root total extract was cytotoxic in pups transplacentally exposed to this plant extract. The present work provides scientific support for the safer use of *J. dioica* in traditional medicine and can also provide information of health effects after transplacental exposure to xenobiotics and contribute to having a better understanding of the plant safety profile.

## Figures and Tables

**Table 1:** MNPCE and PCE values from the pregnant rat study group at different sampling times.

Sampling time (h)	Negative control (*n* = 6)		*J. dioica*: low dose 60 mg/kg (*n* = 6)		*J. dioica*: middle dose 100 mg/kg (*n* = 6)		*J. dioica*: high dose 300 mg/kg (*n* = 6)		Positive control (*n* = 6)	
	MNPCE	PCE	MNPCE	PCE	MNPCE	PCE	MNPCE	PCE	MNPCE	PCE
0	0.22 ± 0.26	40.83 ± 11.60	0.33 ± 0.33	54.00 ± 12.51	0.38 ± 0.38	55.83 ± 16.11	0.13 ± 0.18	47.60 ± 7.50	0.66 ± 0.21	38.17 ± 8.86
			NS^b^	NS^b^	NS^b^	NS^b^	NS^b^	NS^b^	*P*=0.04^b^	NS^b^

24	0.27 ± 0.32	36.67 ± 4.08	0.73 ± 0.54	46.80 ± 4.26	0.33 ± 0.36	51.83 ± 10.18	0.13 ± 0.18	45.60 ± 7.70	0.55 ± 0.54	38.17 ± 3.12
	NS^a^	NS^a^	NS^a^	NS^a^	NS^a^	NS^a^	NS^a^	NS^a^	NS^a^	NS^a^
			NS^b^	NS^b^	NS^b^	*P*=0.002^b^	NS^b^	NS^b^	NS^b^	NS^b^

48	0.33 ± 0.36	36.67 ± 8.35	0.46 ± 0.29	47.60 ± 8.26	0.60 ± 0.57	50.17 ± 6.55	0.33 ± 0.33	38.20 ± 6.38	0.55 ± 0.34	40.00 ± 11.67
	NS^a^	NS^a^	NS^a^	NS^a^	NS^a^	NS^a^	NS^a^	NS^a^	NS^a^	NS^a^
			NS^b^	NS^b^	NS^b^	*P*=0.04^b^	NS^b^	NS^b^	NS^b^	NS^b^

72	0.61 ± 0.57	38.33 ± 7.60	0.53 ± 0.50	44.20 ± 8.58	0.60 ± 0.25	47.17 ± 8.84	0.39 ± 0.36	41.60 ± 11.92	0.55 ± 0.27	34.50 ± 9.39
	NS^a^	NS^a^	NS^a^	NS^a^	NS^a^	NS^a^	NS^a^	NS^a^	NS^a^	NS^a^
			NS^b^	NS^b^	NS^b^	NS^b^	NS^b^	NS^b^	NS^b^	NS^b^

96	0.33 ± 0.51	41.67 ± 10.94	0.46 ± 0.37	48.8 ± 9.03	0.38 ± 0.44	41.17 ± 4.21	0.91 ± 0.95	38.20 ± 12.21	0.60 ± 0.49	18.33 ± 5.68
	NS^a^	NS^a^	NS^a^	NS^a^	NS^a^	NS^a^	NS^a^	NS^a^	NS^a^	*P*=0.007^a^
			NS^b^	NS^b^	NS^b^	NS^b^	NS^b^	NS^b^	NS^b^	*P*=0.001^b^

120	0.66 ± 0.76	39.17 ± 15.72	0.52 ± 0.18	43.20 ± 12.55	0.55 ± 0.45	47.50 ± 10.78	0.73 ± 0.63	34.60 ± 7.02	0.33 ± 0.29	5.83 ± 1.47
	NS^a^	NS^a^	NS^a^	NS^a^	NS^a^	NS^a^	NS^a^	NS^a^	NS^a^	*P*=0.005^a^
			NS^b^	NS^b^	NS^b^	NS^b^	NS^b^	NS^b^	NS^b^	*P*=0.001^b^

Data (‰) are expressed as mean ± standard deviation per group. MNPCE: micronucleated polychromatic erythrocytes/1000 PCE; PCE: polychromatic erythrocytes/1000 TE; TE: total erythrocytes; *n*: sample size, pregnant rats per group; NS: not significant; *J. dioica: Jatropha dioica*.;^a^intragroup significance (repeated measures ANOVA and Bonferroni test post hoc for multiple comparisons);^b^intergroup significance (one-way ANOVA and Dunnett *t*-test post hoc for multiple comparisons).

**Table 2:** MNE, MNPCE, and PCE frequencies from pups of the pregnant rat study group.

Groups	*n*	MNE	MNPCE	PCE
Negative control	6	1.83 ± 0.52	3.67 ± 1.58	653.83 ± 116.69

*J. dioica*: low dose (60 mg/kg)	6	1.35 ± 0.55	3.60 ± 1.52	608.30 ± 88.23
		NS	NS	NS

*J. dioica*: middle dose (100 mg/kg)	6	1.66 ± 0.59	4.28 ± 2.65	609.47 ± 157.84
		NS	NS	NS

*J. dioica*: high dose (300 mg/kg)	6	1.70 ± 0.46	3.73 ± 2.47	521.45 ± 122.76
		NS	NS	*P*=0.001

Positive control: CP (60 mg/kg)	6	11.27 ± 4.21	27.60 ± 10.64	532.47 ± 115.20
		*P*=0.001	*P*=0.001	*P*=0.001

Data (‰) are expressed as mean ± standard deviation per group. *J. dioica* aqueous extract was administered orally to pregnant rats. MNE: micronucleated erythrocytes/1,000 TE; TE: total erythrocytes; MNPCE: micronucleated polychromatic erythrocytes/1,000 PCE; PCE: polychromatic erythrocytes/1,000 TE; *n*: sample size (pregnant rats/6 pups per dam); NS: not significant. Intergroup analysis by means of one-way ANOVA and Dunnet's *t*-test posthoc for multiple comparisons.

**Table 3:** Weight and number of offsprings in new born rats from mothers treated with *J. dioica* during pregnancy.

Study groups	Weight (g)	Number of offsprings
Negative control (*n* = 6)	5.76 ± 0.48	6.43 ± 2.99

*J. dioica*: low dose 60 mg/kg	6.0 ± 0.30	6.17 ± 2.63
	NS	

*J. dioica*: middle dose 100 mg/kg	6.33 ± 0.47	5.43 ± 2.50
	NS	

*J. dioica*: high dose 300 mg/kg	5.56 ± 0.50	7.50 ± 4.32
	NS	

Positive control	4.85 ± 0.48	8.0 ± 2.82
	*P*=001	

Data are expressed as mean ± SD; NS: not significant; *n*: sample size (pregnant rats/6 pups per dam). All study groups were compared with the negative control.

## Data Availability

The raw data required to reproduce these findings cannot be shared at this time as the data also form a part of an ongoing study.

## References

[1] Butler M. S. (2004). The role of natural product chemistry in drug discovery. *Journal of Natural Products*.

[2] Gemelli T. F., da Silva Prado L., Santos F. S. (2015). Evaluation of safety of *Arrabidaea chica* verlot (Bignoniaceae), a plant with healing properties. *Journal of Toxicology and Environmental Health, Part A*.

[3] Richardson A. (2010). *Plants of Deep South Texas: A Field Guide to the Woody and Flowering Species*.

[4] Fresnedo J., Orozco Q. (2013). Diversity and distribution of genus *Jatropha* in Mexico. *Genetic Resources and Crop Evolution*.

[5] Artschwager K. M. (1996). *Healing with Plants in the American and Mexican West*.

[6] Mujumdar A. M., Misar A. V. (2004). Anti-inflammatory activity of *Jatropha curcas* roots in mice and rats. *Journal of Ethnopharmacology*.

[7] Silva-Belmares Y., Rivas-Morales C., Viveros-Valdez E., Cruz-Galicia M. G. d. l., Carranza-Rosales P. (2014). Antimicrobial and cytotoxic activities from Jatropha dioica roots. *Pakistan Journal of Biological Sciences*.

[8] Wong-Paz J. E., Contreras-Esquivel J. C., Rodríguez-Herrera R. (2015). Total phenolic content, in vitro antioxidant activity and chemical composition of plant extracts from semiarid Mexican region. *Asian Pacific Journal of Tropical Medicine*.

[9] Gutiérrez-Tlahque J., Leobardo C., Raya-Pérez J. (2018). Effect of climate conditions on total phenolic content and antioxidant activity of Jatropha dioica Cerv. var. dioica. *Ciencia e investigación agraria*.

[10] Devappa R. K., Makkar H. P. S., Becker K. (2010). Jatropha toxicity-A review. *Journal of Toxicology and Environmental Health, Part B*.

[11] Alanís-Garza B. A., González-González G. M., Salazar-Aranda R., Waksman de Torres N., Rivas-Galindo V. M. (2007). Screening of antifungal activity of plants from the northeast of Mexico. *Journal of Ethnopharmacology*.

[12] Martínez N. (2013). Evaluación del efecto quimioprotector de la decocción de la raíz de *Jatropha dioica* en ratones albinos suizos cepa ICR mediante Ensayo Cometa in vivo. http://dgsa.uaeh.edu.mx:8079/bibliotecadigital/handle/231103/1842.

[13] Badrie N., Schauss A. G. (2010). Soursop (Annona muricata L.) uses: composition, nutritional value, medicinal uses, and toxicology. *Bioactive Foods in Promoting Health: Fruits and Vegetables*.

[14] Oliveira Simone G. D., Nascente P. S., Escareño J. J. H., Carvalho R. V., Piva E., Lund R. G. (2013). Evaluation of anti-*Candida* activity and cytotoxicity of *Jatropha dioica* Cerv. extracts. *African Journal of Microbiology Research*.

[15] Araujo-Espino D. I., Zamora-Perez A. L., Zúñiga-González G. M., Gutiérrez-Hernández R., Morales-Velazquez G., Lazalde-Ramos B. P. (2017). Genotoxic and cytotoxic evaluation of *Jatropha dioica* Sessé ex Cerv. by the micronucleus test in mouse peripheral blood. *Regulatory Toxicology and Pharmacology*.

[16] Bonassi S., Coskun E., Ceppi M. (2011). The Human Micronucleus project on exfoliated buccal cells (HUMN(XL)): the role of lifestyle, host factors, occupational exposures, health status, and assay protocol. *Mutation Research*.

[17] Schmid W. (1975). The micronucleus test. *Mutation Research/Environmental Mutagenesis and Related Subjects*.

[18] Gómez-Meda B. C., Zúñiga-González G. M., Zamora-Perez A., Ramos-Ibarra M. L., Batista-González C. M., Torres-Mendoza B. M. (2004). Folate supplementation of cyclophosphamide-treated mothers diminishes micronucleated erythrocytes in peripheral blood of newborn rats. *Environmental and Molecular Mutagenesis*.

[19] Alcántar-Díaz B. E., Gómez-Meda B. C., Zúñiga-González G. M. (2012). Genotoxic evaluation of pirfenidone using erythrocyte rodent micronucleus assay. *Food and Chemical Toxicology*.

[20] Vanparys P., Deknudt G., Vermeiren F., Sysmans M., Marsboom R. (1992). Sampling times in micronucleus testing. *Mutation Research Letters*.

[21] Zúñiga-González G., Gómez-Meda B. C., Zamora-Perez A. (2003). Induction of micronuclei in proestrus vaginal cells from colchicine and cyclophosphamide treated rats. *Environmental and Molecular Mutagenesis*.

[22] Zúñiga-González G., Torres-Bugarín O., Ramos-Ibarra M. L. (2001). Variation of micronucleated erythrocytes in peripheral blood of Sciurus aureogaster in relation to age: an increment of micronucleated polychromatic erythrocytes after the administration of colchicine. *Environmental and Molecular Mutagenesis*.

[23] Poole T. (1994). *The UFAW Handbook on the Care & Management of Laboratory Animals*.

[24] APA (2011). *APA: The American Psychological Association Guidelines for Ethical Conduct in the Care and Use of Animals*.

[25] Heddle J. A., Cimino M. C., Hayashi M. (1991). Micronuclei as an index of cytogenetic damage: past, present, and future. *Environmental and Molecular Mutagenesis*.

[26] Gómez-Meda B. C., Bañales-Martínez L. R., Zamora-Perez A. L. (2016). Micronucleated erythrocytes in peripheral blood from neonate rats exposed by breastfeeding to cyclophosphamide, colchicine, or cytosine-arabinoside. *BioMed Research International*.

[27] Yang J., Long Y. O., Paquette L. A. (2003). Concise total syntheses of the bioactive mesotricyclic diterpenoids jatrophatrione and citlalitrione. *Journal of the American Chemical Society*.

[28] Zhang L., Gao L., Li Z. (2012). Bio-guided isolation of the cytotoxic terpenoids from the roots of *Euphorbia kansui* against human normal cell lines L-O2 and GES-1. *International Journal of Molecular Sciences*.

[29] Maulik D. (2006). Fetal growth restriction: the etiology. *Clinical Obstetrics and Gynecology*.

[30] Alice C. B., Vargas V. M. F., Silva G. A. A. B. (1991). Screening of plants used in South Brazilian folk medicine. *Journal of Ethnopharmacology*.

[31] Sabandar C., Ahmat N., Mohd F., Sahidin I. (2013). Medicinal property, phytochemistry and pharmacology of several species (Euphorbiaceae): a review. *Phytochemistry*.

[32] Ewing G., Tatarchuk Y., Appleby D., Schwartz N., Kim D. (2015). Placental transfer of antidepressant medications: implications for postnatal adaptation syndrome. *Clinical Pharmacokinetics*.

